# Health reporting on people with a migration background – Selection and definition of (core) indicators

**DOI:** 10.25646/6073

**Published:** 2019-09-18

**Authors:** Susanne Bartig, Alexander Rommel, Annelene Wengler, Claudia Santos-Hövener, Thomas Lampert, Thomas Ziese

**Affiliations:** Robert Koch Institute, Berlin Department of Epidemiology and Health Monitoring

**Keywords:** MIGRATION, PEOPLE WITH MIGRATION BACKGROUND, INDICATORS, HEALTH, HEALTH REPORTING

## Abstract

As part of the project Improving Health Monitoring in Migrant Populations (IMIRA), a (core) set of indicators was developed to describe the health of people with a migration background. This work was underpinned by research into and assessment of relevant data sources in the field of migration and health.

Initially, four fields of action were identified together with a number of associated topics and potential indicators for each of the area’s individual topics. The choice of core indicators was based on (1) a systematic comparison of widely accepted indicator systems, (2) an assessment of public health relevance, (3) comprehensibility and (4) informative value, as well as (5) the availability of (largely) representative data that could properly account for the diversity of the migrant population. The (core) indicator set was finalised using an internal and external indicator development process that involved an interdisciplinary expert panel. This resulted in the selection of 25 core indicators; 41 additional indicators were documented as part of an ‘extended’ indicator set. The (core) set of indicators is to be continually developed in line with the work being undertaken to improve the integration of people with a migration background in the health monitoring conducted at the Robert Koch Institute. In the future, the indicator set is to be incorporated into an overall concept to regular, migration-related health reporting.

## 1. Introduction

Federal Health Reporting regularly provides information about the health of the population in Germany. It uses current representative data to describe temporal developments and to identify health policy areas where action needs to be taken. Evidence-based findings can be used to draw up measures aimed at improving and strengthening the health of the population and assessing the potential impact of these measures [1-3].

People with a migration background account for a significant proportion of the population in Germany: according to the microcensus, almost a quarter of the population in Germany (23.6%) was born either themselves or at least one parent without German citizenship. Almost half of the population with a migration background (48.9%) holds a foreign passport, and more than two-thirds moved to Germany themselves (68.4%) [4]. Through various factors, the cross-border relocation of a person’s permanent centre of life (international migration) [5, 6] influences the life situation of immigrants and the generation born in Germany before, during and after the biographical event of migration [7, 8].

**Info box:**

**Defining indicators and core indicators**

Indicators are empirically measurable values that provide indications about issues that cannot be directly quantified or that are difficult to quantify [15, 16]. In this article, core indicators have high (1) public health relevance, (2) comprehensibility, plausibility and (3) informative value, (4) are used at the national and international level and (5) can be described using a representative data source. Furthermore, they have been identified as particularly relevant by an expert-supported procedure.

Generalisations about the health of people with a migration background cannot reflect the heterogeneity of this population group. The health chances and disease risks vary within the migrant population according to sociodemographic and migration-related factors. As such, reliable data are crucial in order to identify health policy areas where action needs to be taken. However, very few sources of data (currently) exist that include both a representative sample of the migrant population and information about their health [9, 10]. At the 2015 Integration Summit, representatives from politics, migrants’ organisations and the health sector emphasised the importance of ensuring that epidemiology and health reporting regularly consider the health of people with a migration background [11].

The Improving Health Monitoring in Migrant Populations project (IMIRA, project duration 2016-2019), which was conducted at the Robert Koch Institute, aimed to improve the information available on the health of people with a migration background. One of the project's subgoals was the development of a concept for the regular health reporting on people with a migration background [12]. As part of the expansion of health reporting, a (core) set of indicators was developed to describe the health of people with a migration background, which is presented in this article. In the future, the (core) indicator set is to be integrated into Federal Health Reporting’s overall approach and will act as a framework that provides orientation for migration-related health reporting. The following questions were posed during the development of the set of indicators: Which topics and indicators should be taken into account when describing the health of people with a migration background? Which indicators are particularly relevant to migration-related health reporting (see Info box)? Which data sources are suitable for describing these indicators?

Following the principle of monitoring, a limited number of meaningful and measurable core indicators have been selected [13, 14] to provide the most comprehensive overall picture possible of the health situation of people with a migration background. In addition to the core indicators, an ‘extended indicator set’ was also drawn up. In contrast to the core set of indicators, the extended set also includes conceptually relevant indicators for which no reliable data sources could be identified (‘ideal type’ indicators). Therefore, in the context of this article, the umbrella term ‘(core) indicator set’ refers to both the ‘core indicators’ and the ‘extended indicator set’ for describing the health of people with a migration background.

## 2. The conceptual development of the (core) indicator set

**Figure 1:**
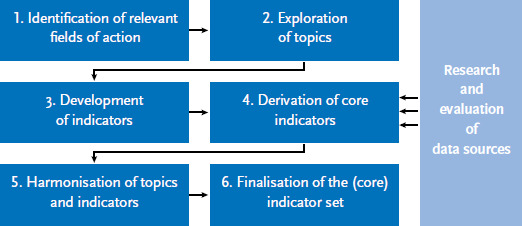
Work steps for the development of a (core) indicator set for migration-related health reporting Source: Own diagram

The following provides more details about the individual steps that were undertaken to develop a (core) indicator set for migration-related Federal Health Reporting. In parallel to the conceptual development of the (core) indicator set, a list of existing data sources (survey and routine data) was compiled before specific criteria were used to review their potential applicability to health reporting on people with a migration background (Figure 1).

The results from the review of the availability of reliable data in the field of migration and health were essential to the selection of the core indicators. Due to their considerable importance for the development of the (core) indicator set, the data-specific challenges faced by migration-related health reporting and the criteria-based selection of data sources are explained in more detail below (see Section 3).

### 2.1 The identification of relevant fields of action and topics (steps 1 and 2)

Based on the World Health Organization’s (WHO's) [17-19] European guidelines on the health of people with a migration background and the existing structure of Federal Health Reporting, four fields of action were identified that constituted the conceptual framework. These were: (1) promoting and strengthening health, (2) promoting and strengthening health-conscious behaviour, (3) promoting health-related resources and reducing risks, and (4) promoting equal access to health care services. Subsequently, nationwide reviews and explanatory approaches in the field of migration and health were systematically assessed in order to identify relevant topics for migration-related health reporting. A provisional selection of topics was made once these topics had been assigned to relevant fields of action. This selection depended on an assessment of a topic’s public health relevance but also took current research into the population with a migration background and the availability of relevant indicators for each topic into account.

### 2.2 The development of the indicators and the derivation of the core indicators (steps 3 and 4)

**Figure 2:**
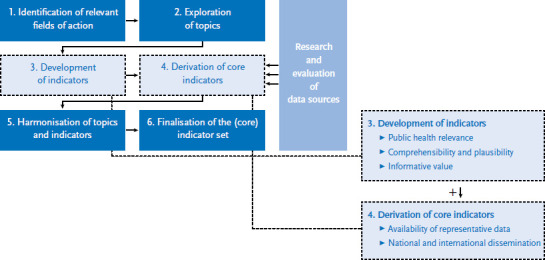
Criteria for the development of indicators and derivation of core indicators Source: Own diagram

In line with the ZWERG guidelines for indicator evaluation (central importance, efficiency, simplicity, timeliness, accuracy) [20], ‘ideal type’ indicators were drawn up for each of the topics. These indicators have to be informative, easy to understand, comprehensible and relevant to the conception of public health policies (Figure 2). Due to the issues related to data availability (see Section 3), the extended set of indicators also includes indicators that cannot currently be described by representative data. In contrast, the existence of reliable data was an essential criterion for the selection of the core indicators (Figure 2). Furthermore, each core indicator was also derived conceptually on the basis of a comparison of established indicator systems that provided for an assessment of how widespread an indicator was at the national and international level. In addition to the Federal Health Reporting and the indicator set used by the Permanent Working Group of the Highest State Health Authorities (AOLG) [21], the review also considered the European Core Health Indicators (ECHI) of the European Commission [22], the Core Health Indicators of the World Health Organization [23], and the core indicators (Health at a Glance) selected by the Organisation for Economic Co-operation and Development (OECD) and the EU [24]. Additionally, the indicators published in the 2018 ‘Report on the health of refugees and migrants in the WHO European Region: no public health without refugee and migrant health’ were taken into account [25].

A research report was produced that includes the public health relevance for each topic, the state of scientific research with regard to the population with a migration background, an overview of the extended indicators and details of the conceptual approach used to derive the core indicators. Furthermore, profiles were compiled for each of the core indicators. In addition to the definition of the core indicator, these include recommendations on possible data sources and a brief outline of the scientific background. These details provided the foundation for the internal and external indicator development process that was used to finalise the (core) indicator set.

The profile drawn up for the prevalence of obesity has been included in the Annex as an example (Annex Table 1). In addition, the research report, which among others includes a description of the criteria-based selection of (core) indicators, is available on the IMIRA website.

### 2.3 Harmonisation and finalisation of the (core) indicator set (steps 5 and 6)

The topics were analysed using a multi-step process: after they had been selected using a project-internal process, the various units at the Robert Koch Institute’s Department of Epidemiology and Health Monitoring also conducted a review of the selected topics. IMIRA’s Advisory Board was then asked to assess the topics for relevance and to select those that should be considered as part of migration-related health reporting. In a written, semi-standardised procedure, the Advisory Board used a 9-point scale ranging from 1 (not relevant) to 9 (highly relevant) to evaluate the relevance of the topics and their determinants for describing the health of people with a migration background. In addition, once a particular field of action had been completed, an opportunity was provided to comment on or add to the topics. The Advisory Board was also informed that the aim of the process was to draw up a limited number of core topics from which a ‘lean’ set of indicators could be derived.

In the evaluation, topics were considered (highly) relevant if at least 60% of the ratings provided by the Advisory Board were in the top categories (7-9). Topics that the Advisory Board deemed relevant for the description of the health of people with a migration background were taken into account in the final selection. However, topics were excluded if they could not be described with data from an adequate source. As such, the final selection of core indicators was based on the results from the internal and external indicator development process as well as on data availability.

## 3. The selection of data sources for the (core) indicator set

Reliable data are essential for identifying health policy areas of action to strengthen the health of people with a migration background. However, the data situation is still inadequate [10, 26]. Only a few data sources are available that provide a comprehensive picture of both the health situation and the migration background and permit differentiated analyses of subgroups within the migrant population, such as by country of origin or the length of stay in Germany.

### 3.1 Data-specific challenges in health reporting on people with a migration background

Official statistics and routine health care data (such as billing data) commonly only include information about citizenship, which means that it is impossible to identify specific migrant subpopulations (such as naturalised citizens or ethnic German resettlers) within the data. In addition, due to a lack of information on the social situation, which has already been empirically proven as a relevant influencing factor of health inequalities [27], the analysis of health-related routine data is clearly limited [28, 29]. Since the sole characteristic “migration background (yes vs. no)” is inadequate for analysing health inequalities [30, 31], other migration-related characteristics (e.g. country of birth, length of stay) must also be taken into account in addition to aspects of the social situation. People with a migration background are often underrepresented in (health) surveys; this means that the proportion of migrants in health surveys often does not correspond to their proportion of the population. This can be due to factors such as linguistic barriers in the process of data collection, which can lead to the systematic exclusion of people with a migration background [32-34]. A migration-sensitive study design, therefore, is essential if the population with a migration background is to be better integrated into surveys.

In line with the challenges identified above, data sources were assessed for their possible uses to migration-related health reporting. The following briefly outlines the procedure that was used to select relevant survey data for use in the health reporting on people with a migration background.

### 3.2 A review of existing (health) surveys

A review was first undertaken of all potential data sources. In addition to the surveys conducted as part of the RKI’s health monitoring framework, surveys by other research institutions (such as the Socio-Economic Panel and the microcensus) were also taken into account. As part of another IMIRA subproject, the possibility of using routine data and data from official statistics was examined. In the review, data sources were included which (1) contained health-related information and (2) collected data on migration background or migration-related characteristics (such as country of birth, length of stay in Germany or residency status). In addition, these data sources had to be nationwide surveys of the entire population in order to enable a comparison between the population with and without a migration background and to apply the findings to Germany as a whole (3). The results were harmonised with another review of data sources in the field of migration and health and published [35].

### 3.3 Evaluation of data sources

The data sources identified by the review were evaluated and prioritised using selected criteria. The aim was to describe the health of people with a migration background using data sources that were (1) up-to-date, (2) (largely) representative and (3) enable statements to be made according to subpopulations in order to take account of the diversity of this population group. In order to ensure representativeness, data sources that applied migration-sensitive study designs were prioritised. To ensure representativeness studies that implemented specific measures to reach people with a migration background either during sampling (such as by oversampling people without German citizenship when using population registries), or during the survey itself (such as by using multilingual survey tools) were prioritised. To adequately reflect the heterogeneity of the population with a migration background, it should also be possible to make differentiated statements according to other relevant determinants of the health situation (migration-related and sociodemographic characteristics). A detailed description of the criteria-based assessment of each data source can be found in the research report published on IMIRA’s website.

### 3.4 The selection of data sources and their assignment to specific indicators

A total of 28 nationwide surveys (Annex Table 2) collect data on at least one health-related aspect in addition to characteristics that enable the identification of participants with a migration background [36]. However, there are clear differences between these data sources with regard to target-group representativeness and the opportunities that they offer in terms of differentiated analyses by sociodemographic and migration-related characteristics.

For the adult population, the criteria used for the analysis led to the prioritisation of the microcensus7 [37], the Socio-Economic Panel (SOEP) [38, 39] and the German Health Interview and Examination Survey for Adults (DEGS1) [40]. If it is possible to describe an indicator with data from more than one source, the studies are listed in order of recommended use. The second wave of the German Health Interview and Examination Survey for Children and Adolescents (KiGGS Wave 2, 2014-2017), employed a migration-sensitive study design that led to an almost representative integration of families with a migration background [41]. As such, it was selected as the preferred data source for describing the health of children and adolescents.

The prioritised data sources were compared for each topic, and recommendations were derived for each indicator. The profiles produced for the core indicators contain the corresponding data sources with information on type of data, data holder and periodicity. In addition to the recommended data sources, the profiles also include data sources that could still be used to describe the indicator but that do not apply a migration-sensitive study design.

## 4. Results

66 indicators were selected to describe the health of people with a migration background; 25 of these were defined as core indicators. An overview of the (core) indicators can be found in the Annex (Annex Table 3). Certain indicators are only relevant to children and adolescents (such as early detection examinations), others are only applicable to adults (such as cancer screening in general and cervical cancer screening).

### 4.1 Characteristics used for stratification

Alongside sociodemographic determinants, people with a migration background differ according to diverse migration-related characteristics such as country of birth, migrant generation, length of stay, residency status, their motives for migration, and their German language skills [31, 42]. As such, different health opportunities and disease risks exist within the migrant population, which means that generalising about the health of people with a migration background produces inadequate results. In order to properly account for the diversity of the population with a migration background, analyses undertaken using the (core) indicator framework should be stratified by selected characteristics. In addition to sex, age and socioeconomic status (low, medium, high), analyses should also provide for a differentiated description of migration background (population without a migration background, population with direct experiences of migration, and second-generation migrants). Depending on the health indicator in question and the opportunities that the data offer for analysis, the following extended framework characteristics should be included in addition to country of birth: length of stay, residency status, the motive behind migration and self-assessed German language skills. This is important because aspects such as linguistic and structural barriers, which result from a person’s residency status, can have an impact on their utilisation of health care services [43, 44].

### 4.2 Promoting and strengthening health

‘Self-assessed general health as good to very good’ and the ‘12-month prevalence of chronic diseases in general’ were selected as core indicators to describe the general health of people with a migration background.

In the field of physical health (noncommunicable diseases), the following core indicators (each as self-reported medical diagnosis) have been derived on the basis of the “world’s biggest killers” [45] named by the WHO and taking into account the still inadequate data quality for the population with a migration background: the ‘lifetime prevalence of heart disease, including cardiac insufficiency/heart failure’ and the ‘lifetime prevalence of stroke’ to map cardiovascular diseases, the ‘lifetime prevalence of bronchial asthma’ to map respiratory diseases and the ‘lifetime prevalence of diabetes mellitus’.

The mental health of people with a migration background may be affected by specific psychosocial factors such as uncertainties about residency status, discrimination and traumatic experiences. This particularly applies to refugees. Combined with social disadvantages, migration-specific factors can place multiple burdens on people with a migration background [46-49]. However, in addition to migration-specific burdens, people with a migration background also have particular psychosocial resources that can have a significant impact on their mental wellbeing as well as their ability to cope with stress [50, 51]. In order to describe mental health, ‘lifetime prevalence of a depressive disorder (self-reported medical diagnosis)’ is recommended as the core indicator for adults with a migration background. For children and adolescents, the ‘prevalence of mental health problems in the last six months’ should be used. Furthermore, the use of the ‘lifetime prevalence of anxiety disorders (self-reported medical/psycho-therapeutic diagnosis)’ is recommended.

Infectious diseases are a major cause of morbidity and mortality, particularly in countries with a low standard of living. In addition to the higher prevalence of certain infectious diseases in some countries of origin, both the migration process itself and the conditions in the country of destination (such as shared accommodation) can increase the risk of infectious diseases [52, 53]. However, information about migration background is only available for a limited number of diseases that are subject to mandatory reporting in accordance with the Protection against Infection Act [44, 53]. Data availability, established indicator systems, and the results of the interdisciplinary indicator development process, led to the selection of ‘tuberculosis cases among people born outside of Germany as a proportion of all tuberculosis cases’ as the core indicator of infectious diseases.

As citizenship is the only migration-related characteristic recorded in official statistics of life expectancy and causes of death, no core indicator was defined for mortality. However, against the background of the limited informative value of the available data sources, mean life expectancy at birth, standardised mortality rate, infant mortality, causes of death and deaths due to suicide were selected as extended indicators.

### 4.3 Promoting health-conscious behaviour

Previous research has identified marked differences in the migrant population in terms of dietary behaviour and physical activity [42, 54, 55]. Therefore, the following core indicators were selected to ensure that migration-related health reporting regularly includes descriptions of dietary behaviour and physical activity: the ‘prevalence of sporting inactivity’, ‘daily vegetable consumption’ as a predictor of healthy eating, and the ‘proportion of children who have been exclusively breastfed for at least six months in line with the World Health Organization’s recommendations’. Body mass index (BMI), a ratio of body weight to height (squared), is a measure used to classify underweight, normal weight, overweight and obesity. In addition to the ‘prevalence of overweight’, the ‘prevalence of obesity’ should also be taken into account when describing the BMI of people with a migration background. The current state of research indicates that people with a migration background (especially people who experienced migration themselves) less frequently engage in sporting activities and tend to be more frequently affected by overweight and obesity (children and adolescents with a one-sided as well as two-sided migration background). They, however, consume less frequently alcohol in risky amounts (this applies to first- and second-generation migrants) [42, 56-58]. The ‘prevalence of risky alcohol consumption’ and the ‘prevalence of current tobacco smoking (occasional to daily/regular)’ are recommended as core indicators of substance use/addiction.

### 4.4 Promoting health-related resources and reducing risks

On the one hand, people with a migration background face specific health risks compared to the population without a migration background. On the other hand, people with a migration background have their own specific health-related resources [7, 59]. Alongside a rejection of substance use due to religious beliefs, which depends on the country of birth, and particular dietary habits, the resources that migrant populations may have include a pronounced level of social cohesion within the population itself. ‘A middle to high level of social support’ is a health-related resource [60] and was selected as the core indicator in the field of social and personal resources. Whereas no indicators associated with living and working conditions were classed as (highly) relevant, ‘experiences of discrimination (occasional to frequent)’ was selected as a core indicator of the migration-specific burden faced by members of ethnic minorities.

### 4.5 Promoting equal access to health care services

When utilising the services provided by the health care system, people with a migration background (especially people who experienced migration themselves) may face specific barriers that make equal participation difficult. In addition to obstacles on the individual level, such as a lack of German language skills, experiences of discrimination and structural barriers (such as those associated with residency status) can have an impact on equal access to health care [44, 61-63].

In order to describe the migrant population’s utilisation of preventive services, the ‘full utilisation of the U3 to U9 early detection examinations’ and ‘vaccination rates for the first and second measles vaccinations’ were defined as core indicators for children. The ‘12-month prevalence of cervical cancer screening’ was classed as particularly relevant during the international comparison of established indicator systems and the internal indicator development process. In addition, ‘adherence with the recommended utilisation of dental check-ups’ was also included as a core indicator. The ‘12-month prevalence of the utilisation of outpatient paediatric and general medical services’ was selected to describe the utilisation of health care services by children and adolescents; the ‘12-month prevalence of the utilisation of outpatient services from general practitioners’ was selected in this case for adults.

## 5. Conclusion and outlook

A (core) indicator set was drawn up to describe the health of people with a migration background as part of the Improving Health Monitoring in Migrant Populations (IMIRA) project conducted at the Robert Koch Institute. 25 core indicators were selected through a process based on the conceptual derivation of core indicators and an indicator development process undertaken together with an interdisciplinary panel of experts. In addition, an extended set of indicators was established that documents 41 additional indicators that can be used to conduct a more in-depth analysis of a specific topic. The extended indicator set also contains a number of ‘ideal type’ indicators for which no reliable data source could be found.

A major challenge in the development of the set of (core) indicators was the availability of reliable data that provided for a representative description of people with a migration background and enable differentiated statements according to individual subpopulations. Deficits in the data particularly exist in terms of indicator-based descriptions of health status and the utilisation of health services. Within the framework of the IMIRA project, feasibility studies were carried out into improving the integration of people with a migration background in the health monitoring undertaken at the Robert Koch Institute. The resulting findings will be integrated into the next nationwide interview and examination survey of the adult population (the Health and Nutrition Survey in Germany, gern survey). In addition, a survey sample will also be drawn of people without German citizenship. The aim is to collect representative data for the population with a migration background, with which statements can be made for specific groups within the migrant population [12]. Against this background, it can be assumed that the data available for describing the health of people with a migration background will improve in the future, which also goes hand in hand with the continuous development of the data-driven core indicator set.

In general, the quality of migration-related health reporting depends on the existence of meaningful indicators that are based on representative data. Moreover, these indicators must enable differentiated statements according to relevant sociodemographic and migration-related characteristics in order to take into account the diversity of the population. Finally, health reporting should be undertaken in a sensitive and anti-discriminatory manner; this is particularly important in the current climate of right-wing populist discourse. This entails critical reflection on the terminologies and categories employed by reporting as well as the avoidance of stigmatisation and marginalisation.

## Key statements

- In order to describe the health of the population as comprehensively as possible, a limited number of core indicators are to be used that account for the diversity found within the migrant population.
- The availability of representative data is a crucial criterion in the selection of core indicators.
- The diversity of the population with a migration background should be taken into account when describing health indicators.
- A total of 66 indicators were selected, 25 of which are core indicators.
- The (core) indicator set is to be integrated into an overall concept to regular, migration-related health reporting.

## Appendix

**Annex Table 1:** Example of a profile using the core indicator obesity prevalence Source: Own table

**Indicator: Obesity prevalence**		
Type	Core indicator	
Dimension	Dietary behaviour and physical activity	
Definition	Proportion of adults with a BMI of 30kg/m^2^ or higher; proportion of children and adolescents with a BMI above the 97^th^ percentile of the reference population	
**Data sources**	**Adult population**	**Children and adolescents**
Recommended data sources Data type Periodicity Data owner	Microcensus Primary data (self-reported) 4 years German Federal Statistical Office	KiGGS Primary data (self-reported) 5-6 years Robert Koch Institute
Additional data sources	SOEP, DEGS1	/
Data sources without a migration-sensitive field program	GEDA	HBSC Studie
**Scientific background**		
▶ The prevalence of obesity not only varies by migrant generation but also by country of birth. ▶ In addition, sex and age-specific effects on the prevalence of obesity can be observed within the migrant population.		
**References**		
Kromeyer-Hauschild K, Moss A, Wabitsch M (2015) Referenzwerte für den Body-Mass-Index für Kinder, Jugendliche und Erwachsene in Deutschland: Anpassung der AGA-BMI-Referenz im Altersbereich von 15 bis 18 Jahren. Adipositas 9(3): 123-127 Schenk L, Neuhauser H, Ellert U (2008) Kinder- und Jugendgesundheitssurvey (KiGGS) 2003-2006: Kinder und Jugendliche mit Migrationshintergrund in Deutschland. Beiträge zur Gesundheitsberichterstattung des Bundes. RKI, Berlin Robert Koch-Institut (Ed) (2015) Gesundheit in Deutschland. Gesundheitsberichterstattung des Bundes. Gemeinsam getragen von RKI und Destatis. RKI, Berlin		

BMI = Body Mass Index, SOEP = Socio-Economic Panel, DEGS1 = German Health Interview and Examination Survey for Adults, GEDA = German Health Update, KiGGS = German Health Interview and Examination Survey for Children and Adolescents, HBSC = Health Behaviour in School-aged Children

**Annex Table 2:** Overview of the sources identified during the review of survey data in the field of migration and health (in alphabetical order) Source: Own table

1)	Alcohol survey – Federal Centre for Health Education (BZgA)
2)	Children of Immigrants Longitudinal Survey in Four European Countries (CILS4EU)
3)	Drug affinity among young people in the Federal Republic of Germany – Federal Centre for Health Education (BZgA)
4)	Epidemiological Survey of Substance Abuse in Germany (ESA)
5)	Eurobarometer
6)	European Social Survey (ESS)
7)	European Union Statistics on Income and Living Conditions (EU-SILC)
8)	European Values Survey (EVS)
9)	Gambling survey – Federal Centre for Health Education (BZgA)
10)	Generations and Gender Survey (GGS)
11)	German Ageing Survey (DEAS)
12)	German General Social Survey (ALLBUS)
13)	German Health Interview and Examination Survey for Adults (DEGS1)/German National Health Interview and Examination Survey 1998 (GNHIES98)
14)	German Health Interview and Examination Survey for Children and Adolescents (KiGGS)
15)	German Health Update (GEDA)
16)	German Oral Health study (DMS)
17)	German Survey on Volunteering (FWS)
18)	Health Behaviour in School-aged Children (HBSC)
19)	IAB-BAMF-SOEP Survey of Refugees in Germany – Institute for Employment Research (IAB), Socio-Economic Panel (SOEP) of the German Institute for Economic Research (DIW Berlin) and the Research Centre on Migration, Integration, and Asylum of the Federal Office for Migration and Refugees (BAMF)
20)	IAB-SOEP migration sample – Institute for Employment Research (IAB) and the Socio-Economic Panel (SOEP) of the German Institute for Economic Research (DIW Berlin)
21)	leben in der Arbeit. German cohort study on work, age and health (lidA)
22)	Microcensus
23)	National Educational Panel Study (NEPS)
24)	Panel Analysis of Intimate Relationships and Family Dynamics (pairfam)
25)	Programme for the International Assessment of Adult Competencies (PIAAC)
26)	Socio-Economic Panel (SOEP)
27)	Study on Refugees 2014 by the Federal Office for Migration and Refugees (BAMF)
28)	Survey of Health, Ageing and Retirement in Europe (SHARE)

**Annex Table 3:** Overview of the (core) indicators for the description of the health situation of people with a migration background by topic and field of action Source: Own table

Topic	Indicator	Data source adults/children
**1. Promoting and strengthening health**		
**1.1 General health**		
**Subjective health**	**Self-assessed general health (good to very good)**	**SOEP/KiGGS**
Health-related restrictions in everyday life (A)	Prevalence of health restrictions in everyday life (somewhat to severely limited)	SOEP
Health-related quality of life	Children/adolescents: total score from the Kidscreen 10 questionnaire (good to very good health-related quality of life)	KiGGS
	Adults: total score from the Short Form 36 questionnaire (good to very good health-related quality of life)	DEGS1
**Chronic diseases (general)**	**12-month prevalence of chronic diseases in general**	**SOEP/KiGGS**
**1.2 Physical health**		
**Cardiovascular diseases**	**Lifetime prevalence of heart disease, including cardiac insufficiency and heart failure**	**SOEP**
**Heart disease (A)**	**(self-reported medical diagnosis)**	
**Stroke (A)**	**Lifetime prevalence of stroke (self-reported medical diagnosis)**	**SOEP**
Hypertension (A)	Lifetime prevalence of hypertension (self-reported medical diagnosis)	SOEP
Cancer (A)	Lifetime prevalence of cancer (self-reported medical diagnosis)	SOEP
**Respiratory diseases**		
**Bronchial asthma**	**Lifetime prevalence of bronchial asthma (self-reported medical diagnosis)**	**SOEP/KiGGS**
Chronic obstructive pulmonary disease (COPD)	Lifetime prevalence of chronic obstructive pulmonary disease (self-reported medical diagnosis)	/
**Diabetes mellitus**	**Lifetime prevalence of diabetes mellitus (self-reported medical diagnosis)**	**SOEP/KiGGS**
Allergic diseases	Lifetime prevalence of at least one allergic disease (self-reported medical diagnosis)	DEGS1/KiGGS
Musculoskeletal conditions:		
joint disease (A)	Lifetime prevalence of musculoskeletal disorders (self-reported medical diagnosis)	SOEP
Injuries	Accident rate	Microcensus/KiGGS
**1.3 Mental health**		
**Depressive disorder (A)**	**Lifetime prevalence of a depressive disorder (self-reported medical diagnosis)**	**SOEP**
**Anxiety disorders**	**Lifetime prevalence of anxiety disorders (self-reported medical/psychotherapeutic diagnosis)**	**DEGS1/KiGGS**
Post-traumatic stress disorders	Lifetime prevalence of a post-traumatic stress disorder	/
Dementias	Prevalence of dementia	/
**Mental health problems (C)**	**Prevalence of mental health problems in the last six months (total score from the Strengths and Difficulties Questionnaire)**	**KiGGS**
Attention Deficit Hyperactivity Disorder (ADHD) (C)	Lifetime prevalence of self-reported medical diagnosis of ADHD	KiGGS
**1.4 Infectious diseases**		
**Tuberculosis**	**Tuberculosis cases among people born outside of Germany as a proportion of all tuberculosis cases**	Registration data held in accordance with the Protection against Infection Act
HIV/AIDS	New diagnoses of HIV among people of non-German origin as a proportion of total new HIV diagnoses	Registration data held in accordance with the Protection against Infection Act
Hepatitis A	Hepatitis A cases among people with a migration background as a proportion of all hepatitis A cases	Registration data held in accordance with the Protection against Infection Act
Syphilis (A)	Syphilis cases among people of non-German origin as a proportion of all syphilis cases	Registration data held in accordance with the Protection against Infection Act
Vaccine-preventable childhood diseases (C)	Lifetime prevalence of vaccine-preventable childhood diseases (measles, mumps, rubella, whooping cough)	KiGGS
**1.5 Mortality**		
Life expectancy	Mean life expectancy at birth in years (German vs non-German citizenship)	Official birth and death statistics
Mortality rate	Standardised mortality rate (German vs non-German citizenship)	Official birth and death statistics
Infant mortality (C)	Infant mortality per 1,000 live births (German vs non-German citizenship)	Official birth and death statistics
Causes of death	Five most common causes of death (German vs non-German citizenship)	Causes of death statistics
Suicide	Deaths due to suicide (German vs non-German citizenship)	Causes of death statistics
**2. Promoting health-conscious behaviour**		
**2.1 Dietary behaviour and physical activity**		
Physical (in)activity	Prevalence of adherence to the World Health Organization’s recommendations on physical activity	DEGS1/KiGGS
**Sporting (in)activity**	**Prevalence of sporting inactivity (no sports to very rarely)**	**SOEP/KiGGS**
Fruit consumption	Daily fruit consumption	DEGS1/KiGGS
**Vegetable consumption**	**Daily vegetable consumption**	**DEGS1/KiGGS**
**Breastfeeding (C)**	**Proportion of children who have been exclusively breastfed for at least six months in line with the World Health Organization’s recommendations**	**KiGGS**
Consumption of sugary soft drinks	Daily consumption of sugary soft drinks	DEGS1/KiGGS
**Body Mass Index (BMI)**	**Prevalence of overweight** **Prevalence of obesity**	**Microcensus/KiGGS** **Microcensus/KiGGS**
**2.2 Substance use/addiction**		
**Tobacco use**	**Prevalence of current tobacco smoking (occasional to daily/regular)**	**Microcensus/KiGGS**
**Alcohol consumption**	**Prevalence of risky alcohol consumption (risk-related consumption)**	**SOEP/KiGGS**
Alcohol consumption	Prevalence of heavy episodic drinking	SOEP/KiGGS
Consumption of illicit drugs	Lifetime prevalence of illicit drug use (excluding cannabis use)	Epidemiological Survey of Sustance Abuse/ Drug Affinity Study
Problematic, pathological gambling (A)	Proportion of people with at least problematic gambling behaviour	Gambling Survey
**3. Promoting health-related resources and reducing risks**		
**3.1 Social and personal resources**		
Health literacy	A score of sufficient or above on the overall index in the short version of the survey used in the European Health Literacy Study (HLS-EU-Q16)	/
**Social support**	**A middle to high level of social support**	**DEGS1/KiGGS**
Religiousness	Subjectively rated religiousness (religious to very religious)	SOEP/-
**3.2 Living and working conditions**		
Workload (A)	Prevalence of subjectively perceived health risk of employment	GEDA
Living environment	Subjective impact of noise pollution from road traffic (moderate to very strong)	DEGS1/KiGGS
**3.3 Migration-specific, psychosocial burdens**		
**Experiences of discrimination**	**Experiences of discrimination (occasional to frequent)**	**SOEP/KiGGS**
Sense of belonging/ feeling of not belonging	A feeling of affiliation to the country of origin vs a feeling of affiliation to Germany	SOEP/-
**4. Promoting equal access to health care services**		
**4.1 Utilisation of preventive services**		
Vaccinations (A)	Vaccination rates for seasonal influenza	/
**Vaccinations (C)**	**Vaccination rates for the first and second measles vaccinations**	**KiGGS**
Vaccinations (C)	Prevalence of self-reported full vaccination against human papillomavirus (HPV) Vaccination rates – complete primary immunisation against tetanus	KiGGS KiGGS
**Early detection examinations (C)**	**Full utilisation of the U3 to U9 early detection examinations**	**KiGGS**
Cancer screening (general) (A)	Regular utilisation of cancer screening	DEGS1
**Cervical cancer screening (A)**	**12-month prevalence of cervical cancer screening**	**DEGS1**
Prenatal care (A)	Number of prenatal medical examinations (at least five)	/
**Dental check-ups (C)**	**Adherence with the recommended utilisation of dental check-ups**	**KiGGS**
**4.2 Utilisation of health care services**		
**Outpatient care (paediatrics, general) (C)**	**12-month prevalence of the utilisation of outpatient paediatric and general medical services**	**KiGGS**
**Outpatient care (general) (A)**	**12-month prevalence of the utilisation of outpatient services from general practitioners**	**DEGS1**
Inpatient care	12-month prevalence of the utilisation of hospital treatment	DEGS1/KiGGS
Psychosocial/ psychotherapeutic care	12-month prevalence of the utilisation of services from medical or psychological psychotherapists	DEGS1/KiGGS
Unmet needs	12-month prevalence of unmet care needs	GEDA
Rehabilitative care (A)	Utilisation of a rehabilitation measure in the last three years (inpatient or outpatient)	DEGS1
Geriatric nursing care (A)	Proportion of the total population in need of nursing care (nursing quota)	/

AIDS = Acquired Immune Deficiency Syndrome, DEGS1 = German Health Interview and Examination Survey for Adults, GEDA = German Health Update, HIV= Human Immunodeficiency Virus, KiGGS = German Health Interview and Examination Survey for Children and Adolescents, SOEP = Socio-Economic Panel Bold = core indicators

C = only relevant to children and adolescents, A = only relevant to adults, Bold = core indicators, grey lettering = data sources with a low data quality rating for migration and health issues
